# Application of the time-driven activity-based costing methodology to a complex patient case management program in Portugal

**DOI:** 10.1186/s12913-023-09729-5

**Published:** 2023-07-13

**Authors:** Yasmara Ortet, Joana Seringa, Rui Santana

**Affiliations:** 1 NOVA National School of Public Health, Nova University of Lisbon, Lisbon, Portugal; 2 NOVA National School of Public Health, Public Health Research Centre, Comprehensive Health Research Center, CHRC, Nova University of Lisbon, Lisbon, Portugal

**Keywords:** Case management, Integrated health care, Cost, Multiple chronic conditions

## Abstract

**Background:**

The number of people with chronic diseases has increased globally, as has the number of chronic diseases per person. Faced with this reality, the term “complex patient” is current and actual.

The healthcare costs associated with these patients are high and are expected to increase since most healthcare systems are not yet ready to provide integrated long-term care. In Portugal, several health institutions have made efforts to provide integrated care: case management models have been implemented to complex patients follow-up. However, studies related to cost of these programs are still limited. Therefore, a qualitative investigation was conducted, approaching the design criteria of a case study research, to design a case management program for complex patients and determine its direct costs, following the Time-Driven Activity-Based Costing methodology, in Local Health Unit setting.

**Method:**

The direct costs of providing care to a complex patient involved in a case management program were determined, using the Time-Driven Activity-Based Costing methodology.

A map of the complex patient was drawn, considering a standard flow in the program. Times and costs were allocated to the activities on the map, following Portuguese and international practices of case management models.

**Results:**

A total of 684,45€/year is spent for each new patient in the case management program, of which 452,65€ corresponds to cost of remuneration of professionals involved; and 663,85€/year, for each patient who is in the case management program (over 1 year), where 432,05€ corresponds to cost of the remuneration of the professionals involved.

Follow-up is the most costly phase (80.82%) and where more time is spent (85.62%).

**Conclusion:**

The time spent by professionals and resources involved and the costs associated with each patient were obtained. The economic impact of the analysed activities was not studied, however, according to international authors, when well applied and selected, integrated care models lead to cost reduction and improved health outcomes.

**Supplementary Information:**

The online version contains supplementary material available at 10.1186/s12913-023-09729-5.

## Background

Globally, greater longevity and population growth are observed [1]. Those facts introduce risk factors that can premeditate the development of chronic diseases and increase their absolute number worldwide [1].

Multiple chronic conditions can coexist in a person's health and, thus, influence the complete cycle of healthcare that the person must receive. This type of patients are known as complex patients [1, 2]. According to the definition of the Agency for Healthcare and Quality (AHRQ), complex patients are understood as people with two or more chronic conditions, in which each condition can influence the healthcare provision of another [3] – “*Patients can have an indication for certain medications, but if they have kidney disease or other conditions, these medications may be contraindicated*” [4]*.*

Complex patients have a greater number of potentially preventable health complications, reduced quality of life, and greater consumption of healthcare resources, leading to higher healthcare costs [1, 2].

Healthcare costs, particularly hospital costs, continue to rise [5]. Despite the various measures already implemented (such as budget negotiation, bundled payments, payment for performance, and benchmarking), providers continue to struggle with inefficiencies and difficulties in coordinating the provision of health care throughout the whole cycle, also enhanced by the existing boundaries between care levels [5].

According to the literature, a costing system in the health sector is fundamental to the knowledge of the costs incurred in the provision for financer-provider contracts [6], cost of illness calculations, studies of cost-effectiveness and efficiency of provision by decision-makers [7]. Efficiency models in healthcare advocate the use of a costing system that is viable, reliable, and adequate to the characteristics of the target population [5].

In Portugal, with the aim of increasing the coordination of provision between the primary, secondary and tertiary healthcare levels, on the scope of the National Health Service, in 1999, Local Health Units (LHU) began to be created [8].

LHU are public entities, with business nature and legal personality and administrative, financial, and patrimonial autonomy. Funding is carried out on a per capita risk-adjusted basis to reflect differences in supply and demand from the target population. Data on their costing methods are not available [9, 10].

The high level of care performance of this type of organization is achieved by strengthening the role of primary care center (PCC) as patient and case managers [8]. LHU consist in vertical integrations of healthcare that create and maintain, over time, a common structure between organizations and healthcare levels, aiming the coordination of interdependencies, within the scope of a collective objective—the patient [11].

A successful Portuguese example of healthcare integration is the Alentejo Coast Local Health Unit, with its “Case Management” programme, which is focused on the needs of patients and their families. It faces the conventional model of response to acute illness, in an episodic, reactive way and not focused on chronic conditions. The pillar of this Case Management is the coordination between hospital healthcare and the PCC, based on a multidisciplinary team made up of healthcare professionals. It encourages accessibility and morbidity reduction, creating a single point of contact between the patient and healthcare institutions – the case manager [12]. The case manager is normally a primary care or hospital nurse, who works as part of a multidisciplinary team in the case management program [12]. The purpose of this program is to assess the patient and the family as a whole, considering health, social and economic aspects, and thus draw up an individual plan based on the paradigm of personalized medicine [13] allowing gains in efficiency in medical care provision [12].

For Alentejo Coast Local Health Unit, the results have been clear, including a reduction in the decompensation of chronic patients, and consequently, a 66,6% to 77,1% decrease in visits to the emergency department, a 52,4% reduction in hospital admissions and a lower consumption of consultations [13, 14]. The program showed savings of €2.558 in costs per equivalent patient/year [14].

As in the previous example, Matosinhos LHU also operates its services through a case manager with its “Support team for complex chronic patients” program. The program had positive results too in this institution, with: a reduction of 55,6% in the number of hospitalizations for urgent episodes if we consider the same period of the previous year or 63% if we consider the period immediately before the integration of the program; 42,3% reduction in the number of hospitalization episodes if we consider the same period of the previous year or 65,4% if we consider the period immediately before the integration of the program [15].

There is still little research on the determination of costs in LHU. In Portugal, there is no legal framework for costing in of vertical integration healthcare context, as in hospitals example that has the Analytical Accounting Plans for Hospitals of ministry of health [11]. According to the research carried out, in Portugal, although there are healthcare integration projects, no specific costing method is being applied aiming the complex patients [11].

Time-Driven Activity-Based Costing (TDABC) methodology, recommended for integrated care models, calculates the cost per care activity, pathology and time, enabling the determination of precise estimations of the cost of each activity throughout the entire provision cycle to produce a certain result in health [6, 9, 16].

Thus, this investigation proposes the design of a case management program for complex patients and the determination of its direct costs, following the TDABC methodology, in LHU setting.

## Methods

To respond the defined objectives of this study, the methods followed the steps of TDABC methodology [17].

**Fig. 1:**
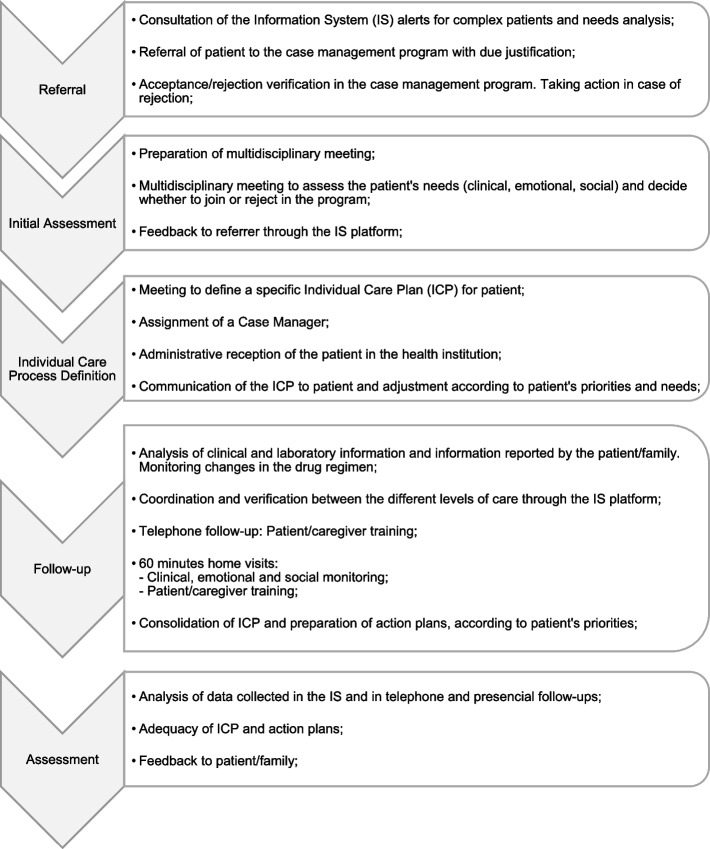
Flow map of a standard complex patient in a case management program, in an LHU [12, 18]. IS—Information System; ICP—Individual Care Plan

To design the flow map of a case management program for complex patients, the following procedures were carried out:

- i - Identification of all key activities carried out throughout the entire cycle of standard care of a complex patient, for one year;
- ii - Identification of direct resources involved in providing care;
- iii - Flow map obtention - Fig. 1

In order to determine the direct costs per complex patient in the case management program, applying the TDABC methodology, the following procedures were carried out:

- i - Obtaining time estimations for each activity and resources used, based on a research article, You E. C. et al. [19], and an LHU Case Management Technical Sheet [12].
- ii - Estimation of the cost of all direct resources involved in the provision of care, using Portuguese Government documents as a reference, allocated according to the time of each activity and resource involved.
- iii - Calculation of the total cost of providing care.

Microsoft® Excel® for Microsoft 365 MSO (version 2205 Build 16. 0. 15,225. 20,172) 64-bit was used.

The perspective of this investigation is the Portuguese National Health Service, more specifically the health care provider LHU.

For confirmation of the data and better knowledge of the context, experts from an LHU were consulted.

- 1 - Complex patient flow-map, in a case management program in a LHU and identification of key activities carried out throughout the care cycle

The flow map is about the standard course of a complex patient in a case management program, in a LHU. That is, due to the heterogeneity of care provided through the heterogeneity of the complex patient's needs, only the standard course to which all selected complex patients are submitted was considered.

The map was built based on adaptations of the model used in a LHU, described in “Case Management Program” [12], and the methods used by *Tortajada S. *et al*.* [18] study. This map is presented in Fig. 1 and Supplementary material 1.

- 2 - Human resources involved and time allocated

For standardization purposes, were always considered 35 work hours per week and 140 work hours per month as the basis for all professionals involved, public sector workers.

For the implementation of the program, various professional groups were considered: Physicians (Senior Internist, Junior Internist, General and Family Medicine), Nurses (hospital and PCC), Social Worker and Operational Assistant.

The choice of these professionals was based on what is being done in an LHU Case Management Program, reported in its technical sheet [12]. As the information available from this LHU is related to the inclusion of 80 patients in the program, for approaching reasons and standardization purposes, this study was adapted to obtain data for “per patient”.

Supplementary material 2 shows the phases and resources spent per complex patient in their standard path in the case management program. It was obtained by gathering information from a LHU technical sheet [12], from the methods used in the study by *Tortajada S. *et al*.* [18], resources from *You E. C. *et al*.* [19] and details provided by experts. The process was outlined with multiple phases: referral, initial assessment, individual care plan definition, follow-up, assessment and two more phases corresponding to undifferentiated actions.

- 3. Time allocated to the different flow map phases

For purposes of time and cost allocation, according to the article *You E. C. *et al*.* [19], two additional phases were contemplated ("Other functions related to the program" and "Other functions not related to the program"), corresponding to non-specific activities, which may arise and have to be carried out by the professionals involved.

To obtain times allocated to each phase in the case management program, the article *You E. C. *et al*.* [19] was used as a reference.

- 4. Time allocated by human resources to flow map different phases

At each phase, the professionals involved were identified, as shown in Supplementary material 1.

From the previous steps, it is known the time that each phase spends in the program. And, the time allocation of each professional to the program is also known. From here, the time allocation of each professional to each phase was determined by crossing the data.

To 100% of “Time allocated to different flow map phases” [19], 40 min were added, referring to the referral phase and 5 min of participation by the Operational Assistant.

- 5. Human resources costs

The costs of professionals involved were based on the 2022 Portuguese Public Administration Remuneration System [20]. The price/hour of professionals was calculated, always taking as reference 35 weekly hours and 140 monthly hours as the base working time for all the professionals involved, public sector workers. For purposes of standardization, all salaries considered were the average salary range, without an exclusivity work contract.

- 6. Other resources involved in the flow-map phases

At all phases, it was considered that information system (IS), technological infrastructure and other necessary infrastructure resources were available within health units, where they are already implemented and used by teams at different care levels. Thus, these resources were disregarded in the mappings when the milestone took place within the facilities of health units.

Since follow-up phase is the only that involves the work of professionals outside health units, it was also the only phase where “Other resources involved” were associated, as shown in Table 3. These resources are directly linked to the exercise of activities outside health units and trigger the need for mobile devices to control and maintain health.

- For limited-use devices, it was assumed that their lifetimes are 3 years and their use divided among 35^1^ patients involved in the program (by case manager, in one year).
- For the transport subsidy, journeys of 100 kilometers were considered;

- 7. Times and costs allocated, by the resources involved, in different flow map phases

At this stage of investigation, all previously presented elements were crossed. To achieve a summary table, formulas were applied to obtain conclusive data that were easy to interpret—presented at Supplementary material 2.

The article used as a basis for calculating the time allocated in each phase (*You E. C. *et al*.* [19]) does not include the time allocated for home visits in follow-up phase. Thus, in this investigation, with the confirmation of LHU experts, 60 min were added for home visits and 90 min for home visits dislocations.

## Results

Results of this investigation were calculated using Microsoft® Excel® for Microsoft 365 MSO (version 2205 Build 16.0.15225.20172) 64-bit.

Each step reported in the Methods represents the calculations performed and Results obtained.

- 1 - Complex patient flow map, in a case management program in a LHU and identification of key activities carried out throughout the care cycle

The results were based on the standard complex patient flow map described in Fig. 1 and Supplementary material 1.

- 2 - Human resources involved and time allocated

The choice of these professionals was based on what is being done in a LHU case management program, reported in its technical sheet [12] and presented at Table 1.

- 3. Time allocated to the different flow map phases

**Table 1:** Time allocated by the human resources involved [12]

Health unit	Professionals involved		Allocation per month per patient	
Hospital	1 senior internist physician		0,2500%	base time
	1 junior internist physician		0,7500%	base time
	1 nurse		0,7500%	base time
	1 social worker		0,0625%	base time
PCC (for each unit)	1 general physician	(1st element)	0,2500%	base time
	1 general physician	(2nd element)	0,0885%	base time
	1 nurse	(1st element)	0,6250%	base time
	1 nurse	(2nd element)	0,3385%	base time
Any level	1 Nurse		0,4762%	base time
	1 operation assistant		0,0595%	base time

*PCC* Primary care center

The time and costs obtained in this investigation, resulting from compilation of *You E. C. *et al*.* [19], LHU technical sheet [12] and the contribution of LHU experts, are described in Supplementary material 2.

- 4. Time allocated by human resources to flow map different phases

Knowing the time allocation of professionals and of each phase, per month in the program, it was possible to determine the allocation of each professional involved in each phase—Supplementary material 2.

In line with what was saw in calculations made in “Human resources involved” and “Time allocated to different flow map phases”, nurses case managers and hospital nurses, in phases in which they participate, are the ones who dedicate most time by program phase, among other professionals, thus validating the central role of nurses in building a case management program.

- 5. Human resources costs

All professionals’ remunerations considered, presented at Table 2, were the average salary position of remuneration table. Thus, the remuneration values of professionals may not correspond to the actual Portuguese practice, as confirmed by LHU experts however, for purposes of standardization, it was necessary to resort to this step. The results related to costs of professionals (Supplementary material 2) may be overestimated.

- 6. Other resources involved in flow map phases

**Table 2:** Costs of professionals involved

Profession	Base salary for 35 h a week	Value considered	Data origin	Price/Time
Senior internist physician	€2.658,02	Value of the average remuneration position of a Graduate Assistant, full-time 35 h, salary level of the single remuneration table (42 and 43);	Public Administration Remuneration System 2022 [20]	€18,99
Junior internist physician	€ 2.110,78	Average remuneration of Assistant, full time of 35 h, salary level of the single remuneration table (32 and 33);		€ 15,08
General physician	€2.658,02	Value of the average remuneration position of a Graduate Assistant, full-time 35 h, salary level of the single remuneration table (42 and 43);		€18,99
Nurses (hospital, PCC, any level)	€ 1.997,60	Value of the average remuneration position of a non-specialist nurse, remuneration position 5, salary level of the single remuneration table (30);		€14,27
Social worker	€2.258,15	Value of the average pay scale for Higher Technician, pay grade 7, pay level of the single pay scale 35;		€16,13
Operational assistant	€705,00	Value of the average remuneration position for an Operational Assistant, remuneration position 4, salary level of the single remuneration table (4);		€5,04

*PCC* Primary care center

Follow-up phase was the only one to have “Other resources involved” associated. As described in Table 3, in Follow-up, the cost of acquiring a mobile phone with internet access, necessary for actions of this phase, was 200€ per case manager. Considering that the useful life of the equipment was given as 3 years and each case manager has 35 patients per year under his care, the cost for using this resource in each home visit is 0,32€.

**Table 3:** Other resources involved

Phase	Action	Activity			Resources involved	No	Cost		Assumed useful life of the resource (in years)	Cost per resource, per activity, per patient
Follow-up	Remote, physical and/or telephone monitoring of healthcare provided at different levels	60 min home visits: - Clinical, emotional and social monitoring -Patient/caregiver training	6 times/year	For proactive follow-up, at least 6 annual visits were assumed	mobile telephone	1	€ 200,00	Cost considered for purchase of resource	3	€0,32
					Transportation	1	€0,36	Per kilometer		€ 36,00
					Mobile parameter monitoring kit	1	€959,40	Cost considered for purchase of resource	3	€1,52
					Mobile computer with VPN	1	€ 500,00	Cost considered for purchase of resource	3	€0,79
										€38,63
								Total follow-up costs, for each home visit, per patient		

*VPN* Virtual Private Network

For home visits dislocation, the transport subsidy considered was 0,36€/Km [21], and for reporting purposes, were considered trips of 100 km (two-ways).

Also, the mobile monitoring kit was estimated 3 years of useful life. Each unit costs 959,40€ [22], leading to a cost per use of 1,52€. This kit corresponds to medical devices with integration and automatic registration in IS: scale, blood pressure meter, oximeter, digital thermometer and portable electrocardiogram.

A laptop with VPN (Virtual Private Network) is also necessary. With 3 years of useful life, it costs 0,79€ per use.

- 7. Times and costs allocated, by the resources involved, in different flow map phases

This calculation step is the intersection of all the steps performed previously. It is the compilation of all the information of the resources considered in this investigation.

The sum of time spent by all professionals, per year, per patient entering the program is 40,49 h; and 39,31 h/year per patient that is in the program over 1 year.

For each patient entering a case management program is spent 684,45€/year, of which 452,65€ corresponds to costs of salaries of professionals involved; and 663,85€/year, for each patient who is in a case management program (over 1 year), in which 432,05€ corresponds to costs of salaries of professionals involved.

Follow-up is the costliest phase (80,82%) and where most time is spent (85,62%), as shown in Table 4.

**Table 4:** Summary of time and costs allocated per phase in a year, per patient

Phase	Time /patient/year (hours)	%	Total time /patient/year (hours)	Cost of professionals /patient/year	%	Cost of other resources involved /patient/ year	%	Total costs /patient/ year	%
Referral	0.6667	1.65%	40.4905	€ 12.66	2.80%			€ 12.66	1.85%
Initial assessment	0.5187	1.28%		€7.95	1.76%			€7.95	1.16%
Individual care process definition	1.1561	2.86%		€16.48	3.64%			€16.48	2.41%
Follow-up	34.6667	85.62%		€ 365.88	80.83%	€ 231.80	100%	€ 597.68	87.32%
Assessment	2.5985	6.42%		€ 37.08	8.19%			€ 37.08	5.42%
Other functions related to the program	0.8803	2.17%		€ 12.56	2.77%			€ 12.56	1.84%
Other functions not related to the program	0.0036	0.01%		€0.05	0.01%			€0.05	0.01%
				Total staff and other resource costs spent in a year for each patient entering a case management program				**€ 684.45**	
				Total staff and other resource costs spent in a year for each patient in a case management program				**€ 663.85**	

The direct costs and calculated times are only related to standard flow of all complex patients, regardless of their chronic conditions and specific health care in the program.

TDABC applications studies have been reporting that indirect costs represent around 26% to 38% and human resources salary 51% to 73% of total costs [23, 24]. In this investigation, only direct costs, essentially human resources, were calculated and accounted for 65% of the total costs. Several authors believe that human resources usually have the greatest weight in a case management program costs [24], greater than 50% [25].

To make Results of this investigation comparable, costs obtained were extrapolated and interpreted as being 51% of the actual cost of the program:

- Extrapolated cost for each patient entering a case management program:  (684,45 €÷ 0,51) = 1.342,06€
- Extrapolated cost for each patient who is in a case management program (over 1 year): (663,85€ ÷ 0,51) = 1.301,66€

## Discussion

The increase in life expectancy has been a global reality, which increases people's exposure to risk factors that trigger chronic diseases [1]. Chronic diseases are a major public health problem, accounting for 60% of all deaths [24]. In the USA, Europe and Spain, the cost of the growing number of chronic diseases represents 75%, 80% and 77%, respectively, of healthcare costs [24].

Studies have revealed that more than 67% of people over the age of 65 have more than one chronic disease [1, 3]. Specifically in Portugal, in 2019, 38.9% of Portuguese people had two or more chronic diseases [26].

Healthcare costs in Europe for chronic diseases are estimated at 700 billion euros per year [27]. It is expected that the incidence of these diseases will continue to increase, asymmetrically with population growth [4]. Thus, the introduction of a new paradigm of integrated care, in all healthcare institutions, is urgent [13, 26], also raising the need to know the incurred costs, through efficient costing methods, since it is also one of the Value-Based Health Care (VBHC) presuppositions [5].

As presented in this investigation, some studies also support that human resources are the biggest cost part of a case management program. Integrated care models aiming home disease management reduce the costs of these programs [24].

In the present investigation, where the model was oriented to diseases self-management and patient empowerment, the extrapolated annual cost of it was 1,301.66€ per patient involved in the program. The extrapolated cost obtained per patient is 2,1 times lower than the base price of hospitalization per DRG costing method (2,759€) [28]. Although this investigation did not consider the cost inherent to different pathologies and care needs in its calculation, it is interesting to note that, on average, a hospitalization is more expensive than the estimated extrapolated cost, direct and annual, of a complex patient in a case management program.

On 2021, one Local Health Unit (Alentejo Coast), with 103,197 registered patients [29], obtained funding of 60.270,192€ [28], i.e. the strict per capita funding was 584,03€. However, knowing that the Portuguese LHU funding takes into account case-mix indices, the real amount paid per capita, by the financier to the provider, was heterogeneous. So, the extrapolated annual cost obtained in this investigation per complex patient is 2,2 times higher than the strict, non-specific funding of Alentejo Coast LHU on 2021.

Within the scope of providing care in an integrated manner, the signed agreements between the financier and the Portuguese health institutions include the integrated financing of programs for specific conditions, such as obesity, diabetes, and chronic obstructive pulmonary disease [28]; which are conditions of common coexistence in complex patients [4]:

- In Surgical Obesity Treatment Program, the funding is defined from 750,67€ to 754,67€/year of follow-up (plus surgery), per patient [28];
- In Treatment of Patients with Continuous Subcutaneous Insulin Infusion Devices Program (patients are defined by the Portuguese National Diabetes Programme), 1.547€/year is paid, by the financier, for each new patient in the program and 1.092€/year^2^ for each patient undergoing follow-up [28];
- In Telemonitoring Program for Chronic Obstructive Pulmonary Disease, the funding per patient is 2.156€/year^3^ [28];

It is important to emphasize that the above-mentioned funding programs are directed to only one chronic condition and, therefore, do not consider the therapeutic complexity and costs associated with patients with the coexistence of two or more chronic conditions.

**Fig. 2:**
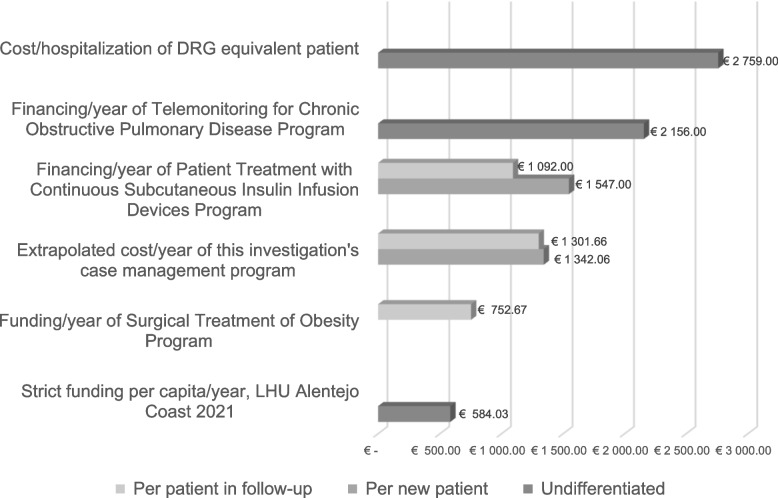
Comparative table of costs and funding. DRG—Diagnosis-related group. LHU – Local Health Unit

To compare the results of this investigation with fundings presented above, the costs obtained were extrapolated and presented in Fig. 2.

Although initially integrated care programs could mean extraordinary investments compared to the conventional care approach, the cost reduction per patient and year in the short term and the health gains for those involved are worth it [27]. *Desmedt M. *et al*.* [27] confirmed it: out of 26 integrated care program studies analysed, 84.6% (*n* = 22) reported positive economic impacts after a short time.

Alentejo Coast LHU reported that with the application of its case management program, it reduced by 57% the number of hospitalizations and 73% of the number of visits to the basic emergency service, of program integrated patients [12]:

- For every 100 hospitalizations, the State (financier) saved 157.263€ [28] (amount obtained from the base price of “Hospitalization and medical and surgical outpatient”);
- For every 100 emergency episodes, the State (financier) saved 3.066€  [28] (amount obtained from the paid amount for “Basic Emergency Service”, reference index 1);

In Portugal, there are few studies about healthcare costing approaches, especially in cases of integrated care for complex patients. Knowing the real costs in health has an impact on several spotlight issues frequently discussed in the context of health policies: financing, efficiencies, risk of strategic decisions, quality of care provision. The advantages gained, at administrative, managing and strategic levels, with knowledge of the real costs of services to complex patients can facilitate a change in the central paradigm of health policies. When the real cost of a complex patient is known, it becomes clear the need to focus strategic decisions on the target population and, perhaps, focus the discussion of health policies not on single disease, but on the complex patient, who has gained so much weight in Portuguese and global populations.

Although the healthcare provision can be major influenced by the available health system resources and even healthcare institution resources, this study results can be used as a guideline for future case managements program implementations.

By knowing the real healthcare costs, resource inefficiencies can also be easily detected and changed, generating an increase in the provision quality, through measures based on the VBHC. Thus, the process is generated as follows: there is theoretical knowledge that helps the decision at the political level that acts with practical measures that have influence at the population and social level.

Due to the unavailability of real costs data, in this investigation it was not possible to determine the difference between the costs of a patient submitted to the conventional healthcare follow-up and the costs of a patient included in this case management program. Also due to a lack of information, the economic impact was not studied. It was not possible to obtain more concrete data.

### Limitations

Below are possible limitations of this investigation:

- Costing was about the common course which all complex patients were submitted into the program. The fact that the different care needs of complex patients was not considered means that the cost obtained per patient is only indicative of a part of their involvement in the case management program and not the whole.
- On professionals’ remunerations were considered the average wage position of the professional class, in the remuneration table. The averages of the salary tables were not considered because they may not be representative. The results may present overestimations of professionals’ remunerations in Portugal.
- When calculating costs, only direct costs were considered. Indirect costs, infrastructure costs and costs with partner entities were not included (as shown in Supplementary materials 1, 2 and 3). Therefore, relevant costs may not have been included in the calculation made. On the other hand, this possible limitation was overcome by calculating the extrapolated cost.
- Finally, economic impact of the case management model was not studied, and no conclusions can be drawn about the cost-efficiency of the proposed interventions or the added value in the health of complex patient.

## Recommendations

Below are opportunities for development from this investigation:

- From the perspective of knowing economic impact of applied case management model, it would be necessary to continue the development of this investigation and exploration of the theme, to determine real gains in health felt in Portugal. Much of literature and studies referenced here believe that most integrated care interventions add value to patients' health and reduce care providing costs.
- It would also be beneficial to continue calculating costs beyond standard course that all complex patients are submitted into the program. That is, specifying costs of most common clusters of comorbidities would be an asset in calculating costs. This would be very well achieved conducting a quantitative study using, retrospectively, follow-up data from a case management LHU program for complex patients. That data, obtained from a close collaboration with an LHU, could be statistically analyzed and separated, creating data groups of the most common clusters of comorbidities of complex patients (obesity, hypertension, dyslipidemia, diabetes, chronic obstructive pulmonary disease and, with lower frequency, conditions related to sleep apnea and kidney disease). This was not conducted in the present investigation for reasons of lack of available data to proceed the methodology and get the quantitative results.
- To the entities responsible for Health in Portugal are recommended to implement more flexible accounting rules that keep up with the versatility of health care for current populations. TDABC costing method would be an added value to the reality of providers, accompanied by a single IS that would facilitate registration of the process and all necessary information to calculate costs. Otherwise, with complex needs growing, how else can provision, costing and correct financing of healthcare be fully studied? How else can we have registered all the costs incurred in maintaining health of populations?

## Conclusion

It is believed that the incidence of chronic diseases, and consequently complex patients, will continue to increase in upcoming years, at a higher rate than population growth. The increase in health costs associated with these patients and the management of multimorbidity are current and future issues that introduce a new paradigm of integrated healthcare.

Although in Portugal, some care integration strategies are already implemented, namely case management, the cost knowledge of these programs are limited. In this investigation, the standard flow of a complex patient was designed in a case management program and direct costs were calculated using TDABC. For each patient in a case management program, an extrapolated cost of 1.301,66€/year was obtained. This amount is lower than Portuguese funding of some chronic disease care programs described in financier-provider contracts. These stated financing programs are directed only to one chronic disease and, therefore, do not consider the heterogeneity of care, thus reinforcing the premise of cost reduction with implementation of integrated care. There is the need to create funding programs dedicated to complex patient, and not just to single chronic diseases.

Effectiveness of interventions and their economic impact were not studied. However, according to several authors, when well applied (essentially consistent with needs of the target population), integrated care programs, namely case management models, generate gains in health and cost reduction, thus being cost-effective in comparison to fragmented conventional care offered by current health systems.

## Supplementary Information

**Additional file 1: Supplementary material 1. **Resources involved in a complex patient case management program, in a LHU [10, 14, 15].

**Additional file 2:Supplementary material 2.** Time and cost allocated, per the resources involved, in the different phases. Calculations performed in a Microsoft® Excel®.

**Additional file 3: Supplementary material 3. **Costs included in the program of this investigation. Direct costs were included.

## Data Availability

The data that support the findings of this study were derived from the following resources available in the public domain: https://www.cnts.min-saude.pt/wp-content/uploads/2019/07/Ficha-Tecnica-Gestao-de-Caso.pdf https://ijic.org/articles/10.5334/ijic.2493 https://bmchealthservres.biomedcentral.com/articles/10.1186/s12913-016-1333-6 https://www.dgaep.gov.pt///upload/catalogo/SRAP_2022_V3.pdf

## Abbreviations

AHRQ: Agency for Healthcare and Quality
ICP: Individual Care Plan
IS: Information System
LHU: Local Health Unit
PCC: Primary Care Center
TDABC: Time-Driven Activity-Based Costing
VBHC: Value-Based Health Care
VPN: Virtual Private Network

## Footnotes

1 In order to ensure follow-up is proactive rather than reactive, ideally a case manager should have a maximum of 35 patients [12].

2 Value includes continuous subcutaneous insulin infusion device and respective consumables, for 12 months of treatment; and all appointments, complementary diagnosis check-ups and therapy related to the regular follow-up of eligible patients, following a defined protocol.

3 Value includes telemonitoring devices.
