# Supplementary material for: Application of the time-driven activity-based costing methodology to a complex patient case management program in Portugal
Source: BMC Health Serv Res. 2023 Jul 13;23:752. doi: 10.1186/s12913-023-09729-5 (PMC10347829; doi:10.1186/s12913-023-09729-5)
Supplement: Supplementary file 1 — Additional file 1: Supplementary material 1. Resources involved in a complex patient case management program, in a LHU [10, 14, 15]. [file 12913_2023_9729_MOESM1_ESM.docx]

Supplementary material 1 - Resources involved in a complex patient case management program, in a LHU (10,14,15).

| Phase | Action | Activity | | Professionals involved | N | Other resources involved (minimum) |
| --- | --- | --- | --- | --- | --- | --- |
| Referral | Referral of the complex patient to the case management program | Consultation of IS alerts for complex patients and analysis of needs | Unique periodicity in the program | Health professional (any level) | 1 | IS platform Email  Telephone |
|  |  | Patient referral to case management program with due justification | Unique periodicity in the program | Health professional (any level) | 1 |  |
|  |  | Acceptance/rejection verification in the case management program. Action taken in case of rejection.  - occurs after the "Initial assessment" | Unique periodicity in the program | Health professional (any level) | 1 |  |
| Initial assessment | Evaluation of the criterias to join the program | Preparation of the multidisciplinary meeting | Unique periodicity in the program | Nurse | 1 | IS platform |
|  |  | Multidisciplinary meeting to assess the patient's needs (clinical, emotional, social) and decide whether to join or reject the program | Unique periodicity in the program | Senior internist physician | 1 | IS platform Meeting room |
|  |  |  |  | Junior internist physician | 1 |  |
|  |  |  |  | General physician | 1 |  |
|  |  |  |  | Nurse (hospital) | 1 |  |
|  |  |  |  | Nurse (PCC) | 1 |  |
|  |  |  |  | Nurse (PCC-case manager) | 1 |  |
|  |  |  |  | Social worker | 1 |  |
|  |  | Feedback to the referrer through the IS platform | Unique periodicity in the program | Nurse (PCC-case manager) | 1 | IS platform |
| Individual care plan definition | Risk stratification and definition of a specific intervention plan, together with the patient/family | Meeting to define a specific ICP for the patient | At this point and annual meeting to review | Senior internist physician | 1 | Meeting room  IS platform  Excel |
|  |  |  |  | Junior internist physician | 1 |  |
|  |  |  |  | General physician | 1 |  |
|  |  |  |  | Nurse (hospital) | 1 |  |
|  |  |  |  | Nurse (PCC) | 1 |  |
|  |  |  |  | Nurse (PCC-case manager) | 1 |  |
|  |  |  |  | Social worker | 1 |  |
|  |  | Assignment of a case manager | Unique periodicity in the program | Senior internist physician | 1 | IS platform Meeting room |
|  |  |  |  | Junior internist physician | 1 |  |
|  |  |  |  | General physician | 1 |  |
|  |  |  |  | Nurse (hospital) | 1 |  |
|  |  |  |  | Nurses (PCC) | 1 |  |
|  |  |  |  | Nurse (PCC-case manager) | 1 |  |
|  |  |  |  | Social worker | 1 |  |
|  |  | Administrative reception of the patient in the health institution | During the activity below | Operational assistent | 1 | IS platform Admission room |
|  |  | Communication of ICP to the patient and adjustment according to patient's priorities and needs | At this point and annual meeting to review | Nurse (PCC-case manager) | 1 | Doctor's office  IS platform  Excel |
|  |  |  |  | Patient/family |  |  |
| Follow-up | Remote, physical and/or telephone monitoring of healthcare provided at different levels | Analysis of information from clinical and laboratory IS and those reported by the patient/family  Follow-up of changes in medication regimen | Every 15 days ~ 24 times a year  This activity was assumed to be before "Telephone follow-up" | Nurse (PCC-case manager) | 1 | IS platform  Patient/family records |
|  |  | Coordination and verification between different levels of care through the IS platform | Every 15 days ~ 24 times a year  This activity was assumed to be before "Telephone follow-up" | Nurse (PCC-case manager) | 1 | IS platform Telephone |
|  |  | Telephone follow-up:  - Patient/caregiver training | Every 15 days ~ 24 times a year | Nurse (PCC-case manager) | 1 | IS platform Telephone |
|  |  |  |  | Patient/family |  |  |
|  |  | 60 minutes home visits:  - Clinical, emotional and social monitoring - Patient/caregiver training | For proactive follow-up, at least 6 annual visits | Nurse (PCC-case manager) | 1 | 1 mobile telephone  Transportation  1 mobile parameter monitoring kit  1 mobile computer with VPN |
|  |  |  |  | General physician | 1 |  |
|  |  |  |  | Patient/family |  |  |
|  |  | Travel for home visit in an single transport - (round trip) 1h30 | During home visits | Nurse (PCC-case manager) | 1 |  |
|  |  |  |  | General physician | 1 |  |
|  |  | Consolidation of ICP and preparation of action plans, according to priorities for the patient | During home visits | Nurse (PCC-case manager) | 1 | IS platform Excel |
|  |  |  |  | Patient/family |  |  |
| Assessment | Evaluation of the patient's clinical, emotional and social evolution | Analysis of data collected on the IS platform and in telephone and presential follow-ups | Monthly evaluation | Nurse (PCC-case manager) | 1 | IS platform |
|  |  | Adequacy of ICP and action plans |  | Nurse (PCC-case manager) | 1 | IS platform Excel |
|  |  | Feedback to patient/family | Monthly evaluation: during an "Telephone follow-up" | Nurse (PCC-case manager) | 1 | IS platform Telephone |
|  |  |  |  | Patient/family |  |  |
| Other functions related to the program | They were assumed to be actions, not included in those reported, to the program to work |  | Monthly action | Nurse (PCC-case manager) | 1 |  |
| Other functions not related to the program | They were assumed to be necessary actions, not included in those reported. |  | Monthly action | Nurse (PCC-case manager) | 1 |  |

IS - Information System. PCC – Primary Care Center.
