# Supplementary material for: Application of the time-driven activity-based costing methodology to a complex patient case management program in Portugal
Source: BMC Health Serv Res. 2023 Jul 13;23:752. doi: 10.1186/s12913-023-09729-5 (PMC10347829; doi:10.1186/s12913-023-09729-5)
Supplement: Supplementary file 2 — Additional file 2:Supplementary material 2. Time and cost allocated, per the resources involved, in the different phases. Calculations performed in a Microsoft® Excel®. [file 12913_2023_9729_MOESM2_ESM.docx]

Supplementary material 2 - Time and cost allocated, per the resources involved, in the different phases. Calculations performed in a Microsoft® Excel®.

| Phase | Action | Activity | | | Professionals involved | | Time/patient | | Time/patient in a year (hours) | | Price/hour of professionals | Professional cost | Professional cost in one year | | | Other resources involved | Cost of other resources involved  / patient | |
| --- | --- | --- | --- | --- | --- | --- | --- | --- | --- | --- | --- | --- | --- | --- | --- | --- | --- | --- |
|  |  |  | periodicity in a year | |  | N | (hours) | (minutes) |  | % |  |  |  |  | % |  | per activity | in a year |
| Referral | Referral of the complex patient to the case management program | Consultation of IS alerts for complex patients and analysis of needs | 1 | Unique periodicity in the program | Health professional (any level) | 1 | 0.1667 | 10,0000 | 0.6667 | 1.65% | €18.99 | €3.16 | €3.16 | € 12.66 | 2.80% | IS platform  Email  Telephone |  |  |
|  |  | Patient referral to case management program with due justification | 1 | Unique periodicity in the program | Health professional (any level) | 1 | 0.3333 | 20,0000 |  |  | €18.99 | €6.33 | €6.33 |  |  |  |  |  |
|  |  | Acceptance/rejection verification in the case management program. Action taken in case of rejection - occurs after the "Initial Assessment" | 1 | Unique periodicity in the program | Health professional (any level) | 1 | 0.1667 | 10,0000 |  |  | €18.99 | €3.16 | €3.16 |  |  |  |  |  |
| Initial assessment | Evaluation of the criteria to join the program | Preparation of the multidisciplinary meeting | 1 | Unique periodicity in the program | Nurse | 1 | 0.0331 | 1.9886 | 0.5187 | 1.28% | € 14.27 | €0.47 | €0.47 | €7.95 | 1.76% | IS platform |  |  |
|  |  | Multidisciplinary meeting to assess the patient's needs (clinical, emotional, social) and decide whether to join or reject the program | 1 | Unique periodicity in the program | Senior internist physician | 1 | 0.0442 | 2.6515 |  |  | €18.99 | €0.84 | €0.84 |  |  | IS platform Meeting room |  |  |
|  |  |  |  |  | Junior internist physician | 1 | 0.1326 | 7.9545 |  |  | € 15.08 | € 2.00 | € 2.00 |  |  |  |  |  |
|  |  |  |  |  | General physician | 1 | 0.0442 | 2.6515 |  |  | €18.99 | €0.84 | €0.84 |  |  |  |  |  |
|  |  |  |  |  | Nurse (hospital) | 1 | 0.1105 | 6.6288 |  |  | € 14.27 | €1.58 | €1.58 |  |  |  |  |  |
|  |  |  |  |  | Nurse (PCC) | 1 | 0.0442 | 2.6515 |  |  | € 14.27 | €0.63 | €0.63 |  |  |  |  |  |
|  |  |  |  |  | Nurse (PCC-case manager) | 1 | 0.0663 | 3.9773 |  |  | € 14.27 | €0.95 | €0.95 |  |  |  |  |  |
|  |  |  |  |  | Social worker | 1 | 0.0105 | 0.6313 |  |  | € 16.13 | €0.17 | €0.17 |  |  |  |  |  |
|  |  | Feedback to the referrer through the IS platform | 1 | Unique periodicity in the program | Nurse (PCC-case manager) | 1 | 0.0331 | 1.9886 |  |  | € 14.27 | €0.47 | €0.47 |  |  | IS platform |  |  |
| Individual care process definition | Risk stratification and definition of a specific intervention plan, together with the patient/family | Meeting to define a specific ICP for the patient | 1 | At this point and annual meeting to review | Senior internist physician | 1 | 0.0811 | 4.8682 | 1.1561 | 2.86% | €18.99 | €1.54 | €1.54 | €16.48 | 3.64% | Meeting room  IS platform  Excel |  |  |
|  |  |  |  |  | Junior internist physician | 1 | 0.2434 | 14.6045 |  |  | € 15.08 | €3.67 | €3.67 |  |  |  |  |  |
|  |  |  |  |  | General physician | 1 | 0.0811 | 4.8682 |  |  | €18.99 | €1.54 | €1.54 |  |  |  |  |  |
|  |  |  |  |  | Nurse (hospital) | 1 | 0.2028 | 12.1705 |  |  | € 14.27 | €2.89 | €2.89 |  |  |  |  |  |
|  |  |  |  |  | Nurse (PCC) | 1 | 0.0811 | 4.8682 |  |  | € 14.27 | € 1.16 | € 1.16 |  |  |  |  |  |
|  |  |  |  |  | Nurse (PCC-case manager) | 1 | 0.0811 | 4.8682 |  |  | € 14.27 | € 1.16 | € 1.16 |  |  |  |  |  |
|  |  |  |  |  | Social worker | 1 | 0.0193 | 1.1591 |  |  | € 16.13 | €0.31 | €0.31 |  |  |  |  |  |
|  |  | Assignment of a case manager | 1 | Unique periodicity in the program | Senior internist physician | 1 | 0.0143 | 0.8591 |  |  | €18.99 | €0.27 | €0.27 |  |  | IS platform Meeting room |  |  |
|  |  |  |  |  | Junior internist physician | 1 | 0.0430 | 2.5773 |  |  | € 15.08 | €0.65 | €0.65 |  |  |  |  |  |
|  |  |  |  |  | General physician | 1 | 0.0143 | 0.8591 |  |  | €18.99 | €0.27 | €0.27 |  |  |  |  |  |
|  |  |  |  |  | Nurse (hospital) | 1 | 0.0358 | 2.1477 |  |  | € 14.27 | €0.51 | €0.51 |  |  |  |  |  |
|  |  |  |  |  | Nurses (PCC) | 1 | 0.0143 | 0.8591 |  |  | € 14.27 | €0.20 | €0.20 |  |  |  |  |  |
|  |  |  |  |  | Nurse (PCC-case manager) | 1 | 0.0143 | 0.8591 |  |  | € 14.27 | €0.20 | €0.20 |  |  |  |  |  |
|  |  |  |  |  | Social worker | 1 | 0.0034 | 0.2045 |  |  | € 16.13 | €0.05 | €0.05 |  |  |  |  |  |
|  |  | Administrative reception of patient in the health institution | 1 | During the activity below | Operational assistant | 1 | 0.0833 | 0.7500 |  |  | €5.04 | €0.42 | €0.42 |  |  | IS platform Admission room |  |  |
|  |  | Communication of ICP to the patient and adjustment according to patient's priorities and needs | 1 | At this point and annual meeting to review | Nurse (PCC-case manager) | 1 | 0.1432 | 8.5909 |  |  | € 14.27 | € 2.04 | € 2.04 |  |  | Doctor's office  IS platform  Excel |  |  |
|  |  |  |  |  | Patient/family |  |  |  |  |  |  |  |  |  |  |  |  |  |
| Follow-up | Remote, physical and/or telephone monitoring of healthcare provided at different levels | Analysis of information from clinical and laboratory IS and those reported by the patient/family  Follow-up of changes in medication regimen | 24 | Every 15 days ~ 24 times a year  This activity was assumed to be before "Telephone follow-up" | Nurse (PCC-case manager) | 1 | 0.0486 | 2.9167 | 34.6667 | 85.62% | € 14.27 | €0.69 | € 16.65 | € 365.88 | 80.83% | IS platform  Patient/family records |  |  |
|  |  | Coordination and verification between different levels of care through the IS platform | 24 | Every 15 days ~ 24 times a year  This activity was assumed to be before "Telephone follow-up" | Nurse (PCC-case manager) | 1 | 0.0486 | 2.9167 |  |  | € 14.27 | €0.69 | € 16.65 |  |  | IS platform  Telephone |  |  |
|  |  | Telephone follow-up: Patient/caregiver training | 24 | Every 15 days ~ 24 times a year | Nurses (PCC-case manager) | 1 | 0.0972 | 5.8333 |  |  | € 14.27 | €1.39 | €33.29 |  |  | IS platform Telephone |  |  |
|  |  |  |  |  | Patient/family |  |  |  |  |  |  |  |  |  |  |  |  |  |
|  |  | 60 minutes home visits:  - Clinical, emotional and social monitoring - Patient/caregiver training | 6 | For proactive follow-up, at least 6 annual visits were assumed | Nurses (PCC-case manager) | 1 | 1.0000 | 60,0000 |  |  | € 14.27 | € 14.27 | € 85.61 |  |  | 1 mobile telephone  Transportation  1 mobile parameter monitoring kit  1 mobile computer with VPN | €38.63 | € 231.80 |
|  |  |  | 6 |  | General and Family Medicine Physician | 1 | 1.0000 | 60,0000 |  |  | €18.99 | €18.99 | € 113.92 |  |  |  |  |  |
|  |  |  | 6 |  | patient/family | AT | AT | AT |  |  | AT | AT | AT |  |  |  |  |  |
|  |  | Travel for home visit in single transport - (round trip) 1h30 | 6 | During home visits | Nurses (PCC-case manager) | 1 | 1.5000 | 90,0000 |  |  | € 14.27 | € 21.40 | € 128.42 |  |  |  |  |  |
|  |  |  |  |  | General physician | 1 | 1.5000 | 90,0000 |  |  | €18.99 | €28.48 | € 170.87 |  |  |  |  |  |
|  |  | Consolidation of ICP and preparation of action plans, according to priorities for the patient | 6 | During home visits | Nurses (PCC-case manager) | 1 | AT | AT |  |  | AT | AT | AT |  |  | IS platform Excel |  |  |
|  |  |  |  |  | Patient/family |  |  |  |  |  |  |  |  |  |  |  |  |  |
| Assessment | Evaluation of the patient's clinical, emotional and social evolution | Analysis of data collected on the IS platform and in telephone and presential follow-ups | 12 | Monthly evaluation | Nurses (PCC-case manager) | 1 | 0.1299 | 7.7955 | 2.5985 | 6.42% | € 14.27 | €1.85 | €22.25 | € 37.08 | 8.19% | IS platform |  |  |
|  |  | Adequacy of ICP and action plans | 12 |  | Nurses (PCC-case manager) | 1 | 0.0433 | 2.5985 |  |  | € 14.27 | €0.62 | €7.42 |  |  | IS platform  Excel |  |  |
|  |  | Feedback to patient/family | 12 | Monthly evaluation: during a "Telephone follow-up" | Nurses (PCC-case manager) | 1 | 0.0433 | 2.5985 |  |  | € 14.27 | €0.62 | €7.42 |  |  | IS platform  Telephone |  |  |
|  |  |  |  |  | Patient/family |  |  |  |  |  |  |  |  |  |  |  |  |  |
| Other functions related to the program | Were assumed to be actions, not included in those reported, for the program to work. |  | 12 | Monthly action | Nurses (PCC-case manager) | 1 | 0.0734 | 4.4015 | 0.8803 | 2.17% | € 14.27 | € 1.05 | € 12.56 | € 12.56 | 2.77% |  |  |  |
| Other functions NOT related to the program | Were assumed to be necessary actions, not included in those reported. |  | 12 | Monthly action | Nurses (PCC-case manager) | 1 | 0.0003 | 0.0178 | 0.0036 | 0.01% | € 14.27 | €0.00 | €0.05 | €0.05 | 0.01% |  |  |  |
| Total time (hours) spent in a year for each patient entering a case management program | | | | | | | | | 40.4905 | Total professionals costs employed in a year for each patient entering a case management program | | | | € 452.65 | Total costs incurred in a year for each patient entering a case management program | | | € 684.45 |
| Total time (hours) spent in a year for each patient in a case management program | | | | | | | | | 39.3051 | Total costs of professionals employed in a year, for each patient who is in a case management program | | | | € 432.05 | Total costs incurred in a year, for each patient who is in a case management program | | | € 663.85 |
|  |  |  |  |  |  |  |  |  |  |  |  |  |  |  |  |  |  |  |

IS - Information System. PCC – Primary Care Center. VPN - Virtual Private Network.
