# Supplementary material for: Application of the time-driven activity-based costing methodology to a complex patient case management program in Portugal
Source: BMC Health Serv Res. 2023 Jul 13;23:752. doi: 10.1186/s12913-023-09729-5 (PMC10347829; doi:10.1186/s12913-023-09729-5)
Supplement: Supplementary file 3 — Additional file 3: Supplementary material 3. Costs included in the program of this investigation. Direct costs were included. [file 12913_2023_9729_MOESM3_ESM.docx]

Supplementary material 3 - Costs included in the program of this investigation. Direct costs were included.
